# Fibroblasts from different body parts exhibit distinct phenotypes in adult progeria Werner syndrome

**DOI:** 10.18632/aging.202696

**Published:** 2021-02-24

**Authors:** Hisaya Kato, Yoshiro Maezawa, Naoya Takayama, Yasuo Ouchi, Hiyori Kaneko, Daisuke Kinoshita, Aki Takada-Watanabe, Motohiko Oshima, Masaya Koshizaka, Hideyuki Ogata, Yoshitaka Kubota, Nobuyuki Mitsukawa, Koji Eto, Atsushi Iwama, Koutaro Yokote

**Affiliations:** 1 Department of Endocrinology, Hematology and Gerontology, Chiba University Graduate School of Medicine, Chuo-Ku, Chiba 260-8670, Japan; 2 Division of Diabetes, Metabolism and Endocrinology, Chiba University Hospital, Chuo-Ku, Chiba 260-8670, Japan; 3 Department of Regenerative Medicine, Chiba University Graduate School of Medicine, Chuo-Ku, Chiba 260-8670, Japan; 4 Gene Expression Laboratory, Salk Institute for Biological Studies, La Jolla, CA 92037, USA; 5 Department of Diabetes and Metabolism, Asahi General Hospital, Asahi-Shi, Chiba 289-2511, Japan; 6 Division of Stem Cell and Molecular Medicine, Center for Stem Cell Biology and Regenerative Medicine, The Institute of Medical Science, The University of Tokyo, Minato-Ku, Tokyo 108-8639, Japan; 7 Department of Plastic, Reconstructive, and Aesthetic Surgery, Chiba University Graduate School of Medicine, Chuo-Ku, Chiba 260-8670, Japan; 8 Department of Clinical Application, Center for IPS Cell Research and Application (CiRA), Kyoto University, Shogoin, Sakyo-Ku, Kyoto 606-8507, Japan

**Keywords:** Werner syndrome, dermal fibroblasts, adipogenesis, chondrogenesis, osteogenesis

## Abstract

Werner syndrome (WS), also known as adult progeria, is characterized by accelerated aging symptoms from a young age. Patients with WS experience painful intractable skin ulcers with calcifications in their extremities, subcutaneous lipoatrophy, and sarcopenia. However, there is no significant abnormality in the trunk skin, where the subcutaneous fat relatively accumulates. The cause of such differences between the limbs and trunk is unknown. To investigate the underlying mechanism behind these phenomena, we established and analyzed dermal fibroblasts from the foot and trunk of two WS patients. As a result, WS foot-derived fibroblasts showed decreased proliferative potential compared to that from the trunk, which correlated with the telomere shortening. Transcriptome analysis showed increased expression of genes involved in osteogenesis in the foot fibroblasts, while adipogenic and chondrogenic genes were downregulated in comparison with the trunk. Consistent with these findings, the adipogenic and chondrogenic differentiation capacity was significantly decreased in the foot fibroblasts *in vitro*. On the other hand, the osteogenic potential was mutually maintained and comparable in the foot and trunk fibroblasts. These distinct phenotypes in the foot and trunk fibroblasts are consistent with the clinical symptoms of WS and may partially explain the underlying mechanism of this disease phenotype.

## INTRODUCTION

Werner syndrome (WS) is a rare autosomal recessive premature aging disorder that begins at a young age with graying and loss of hair and cataracts, followed by accelerated aging symptoms such as diabetes, atherosclerosis, and cancer [1–4]. The median life expectancy is in the mid-50s, and most deaths are due to arteriosclerosis and malignancy [5]. Owing to the founder mutation, a high incidence of WS is observed in Japan [6, 7].

The causative gene is WRN, which is located on chromosome 8 and is involved in DNA replication, DNA repair, and telomere maintenance [8]. WS fibroblasts with deficient or dysfunctional WRN proteins show premature cellular senescence *in vitro* [9]. This phenotype is largely dependent on telomere shortening and can be overcome by telomerase overexpression [10, 11].

WS mimics various symptoms of general aging. However, there are also phenotypes specific to WS, such as refractory skin ulcers with severe pain in the extremities, which affect over 80% of patients and may even result in limb amputation [12]. Common sites for ulceration are the heels, soles, toes, Achilles tendons, and elbows. Painful subcutaneous calcification has been reported to precede skin ulcers [13]. The atrophy of subcutaneous fat and muscle when present in the extremities resembles branches of dried trees, which is diagnosed as sarcopenia [14]. In contrast, there is an accumulation of subcutaneous fat in the trunk [15, 16]. While the skin of the extremities, frequently accompanied by ulcers, is atrophic and tight, that of the trunk retains its elasticity and does not develop ulcers. The underlying mechanism behind these differences remains unknown.

To clarify the relationship between the skin properties and the high prevalence of skin ulcers in the extremities, we established fibroblasts from the skin of the trunk and that of the foot from the vicinity of ulceration in two WS patients.

## RESULTS

### Foot fibroblasts exhibited reduced proliferation compared to the trunk fibroblasts in a telomere-dependent manner

**Figure 1:**
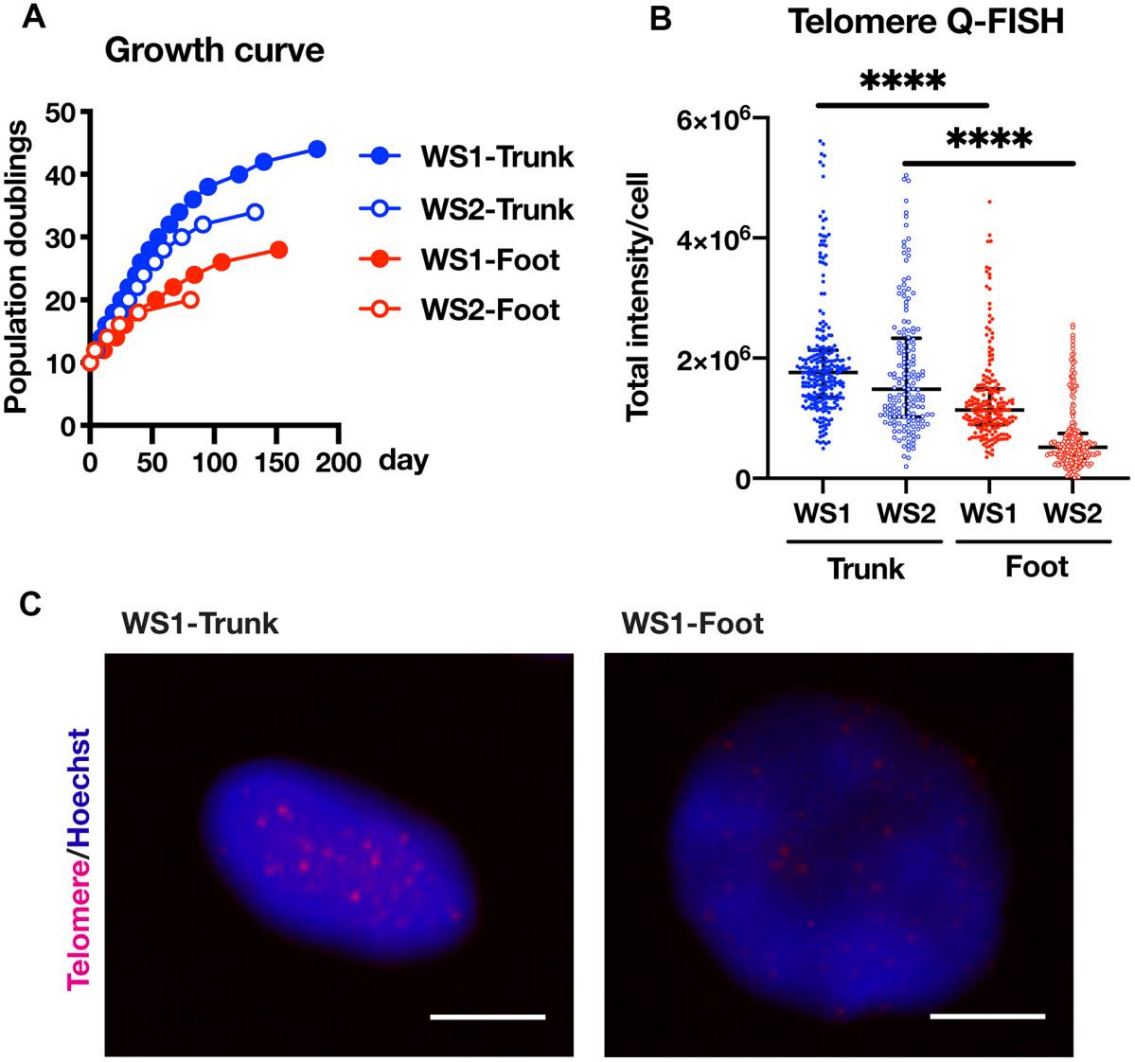
**Foot fibroblasts exhibited reduced proliferative capacity compared to that from the trunk in a telomere length-dependent manner.** (**A**) Growth curves of the fibroblasts from the trunk and foot in two WS patients. (**B**) Telomere length quantification through Q-FISH. Data are median values ± interquartile range of each cell line. More than 150 cells for each cell line were analyzed. For statistical analysis, Mann Whitney test was performed (****p<0.0001). (**C**) Representative image of telomere Q-FISH of WS1. Bar = 10 μm.

In WS, the skin in the extremities atrophies and hardens, and a skin ulcer develops, while there is no obvious abnormality in the trunk skin. Therefore, plastic surgery is often performed to graft skin from the trunk to the ulcer site in order to treat the skin ulcers. In this study, we established fibroblasts from the foot skin (normal skin adjacent to the ulcer) and trunk skin (graft) of two WS patients (WS1 and WS2) who were admitted to our hospital for plastic surgery. We hypothesized that the difference in skin symptoms between the limb and trunk was related to a reduced proliferative capacity of the limb fibroblasts compared to that of the trunk. As expected, the proliferation rate of foot skin-derived fibroblasts was lower than that of the trunk (Figure 1A). In previous reports, the cause of the reduced proliferative potential of WS fibroblasts was explained by shortened telomere length [10, 11]. Consistent with these reports, there was a significant difference in the telomere lengths between the foot and trunk fibroblasts (Figure 1B, 1C and Supplementary Figure 1). These results suggest that the difference in proliferation ability between skin fibroblasts of the foot and trunk is dependent on the telomere length.

### Foot and trunk fibroblasts in WS showed differential expression of genes, especially those involved in embryogenesis and differentiation

**Figure 2:**
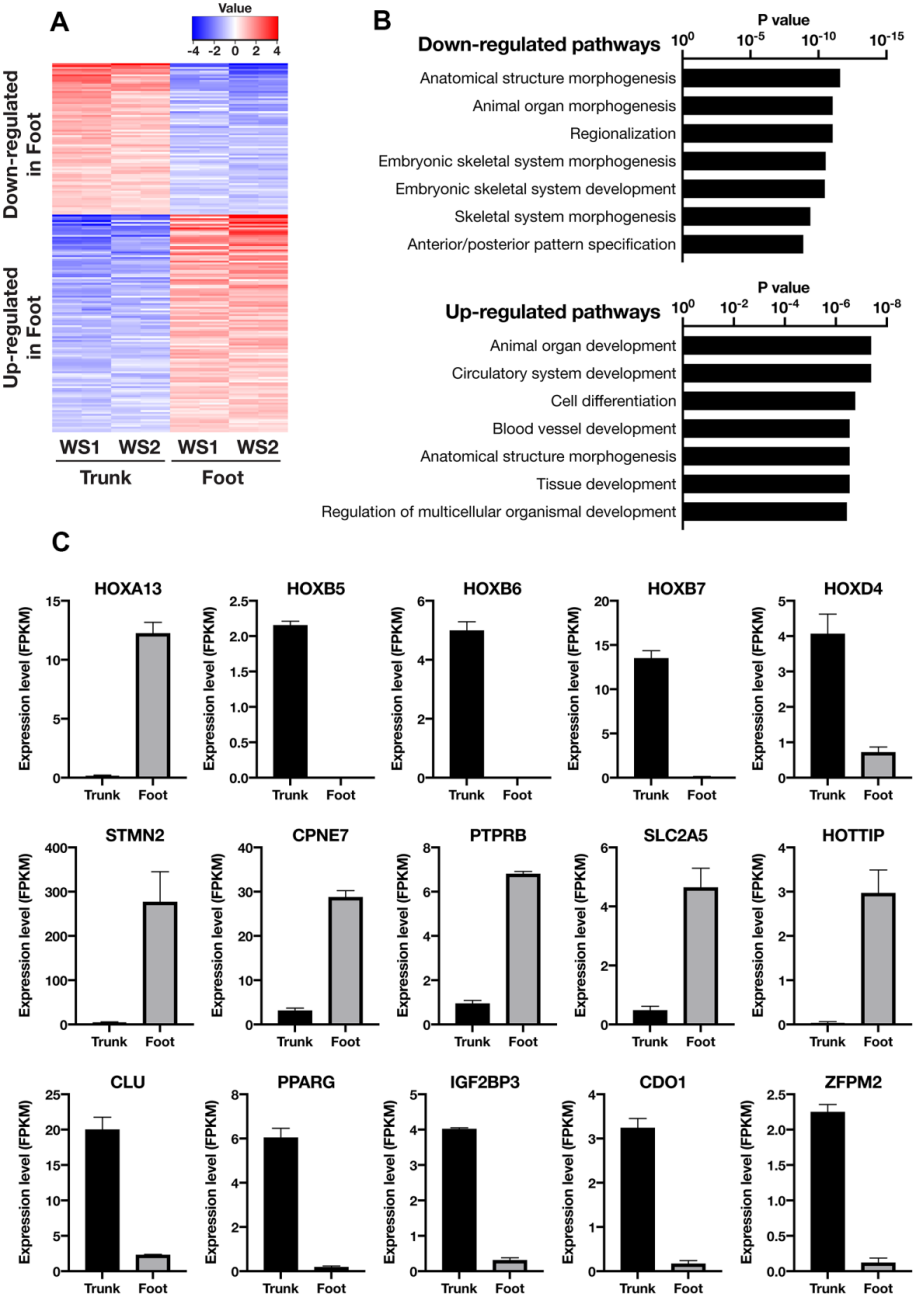
**Transcriptome analysis showed distinct gene expression profiles between the trunk and foot fibroblasts.** (**A**) Heatmap of differentially expressed genes between the trunk and foot. Cutoff: |log2(Foot/Trunk)| > 1 and FDR < 0.05. (**B**) List of the top seven Gene Ontology (GO: biological process) terms and corresponding p values related to Figure 2A. (**C**) Differentially expressed genes specifically involved in embryonic development and mesenchymal cell differentiation. Data are means ± SEM of two patients (technically n=2 in each sample).

Next, transcriptome analysis was performed using RNA sequences to characterize the gene expression profiles of the foot and trunk fibroblasts. In the hierarchical clustering analysis, the WS foot and trunk fibroblasts were classified into different clusters beyond individual differences (Supplementary Figure 2A and Supplementary Table 1). The analysis of differentially expressed genes (DEGs) was performed to identify genes with more than 2-fold differences in expression, and a total of 140 up-regulated and 119 down-regulated genes were identified in the foot (Figure 2A and Supplementary Tables 2, 3). Enrichment analysis of DEGs revealed that their pathways are mainly involved in differentiation and embryogenesis (Figure 2B). Among them, Homeobox A13 (HOXA13) was explicitly expressed in the foot, while Homeobox B5 (HOXB5), Homeobox B6 (HOXB6), Homeobox B7 (HOXB7), and Homeobox D4 (HOXD4) expression were specific to the trunk (Figure 2C), which is consistent with the site-specific gene expression of fibroblasts from normal individuals in previous reports [17, 18]. Intriguingly, the foot fibroblasts showed an elevated expression of genes that relate to the promotion of osteogenesis and suppression of adipogenesis and chondrogenesis, including Stathmin 2 (STMN2), Copine 7 (CPNE7), Protein Tyrosine Phosphatase Receptor Type B (PTPRB), Solute Carrier Family 2 Member 5 (SLC2A5), and HOXA Distal Transcript Antisense RNA (HOTTIP) (Figure 2C) [19–23]. In contrast, in the fibroblasts of the trunk, the expression of the following genes were increased; Clusterin (CLU), Peroxisome Proliferator-Activated Receptor Gamma (PPARG), Insulin-Like Growth Factor 2 MRNA Binding Protein 3 (IGF2BP3), Cysteine Dioxygenase Type 1 (CDO1), and Zinc Finger Protein, FOG Family Member 2 (ZFPM2) (Figure 2C), which are associated with the promotion of adipogenesis or chondrogenesis and suppression of osteogenesis [24–29]. However, regarding senescence-associated genes, no significant differences were observed in the expression of Cyclin-Dependent Kinase Inhibitor 1A (CDKN1A, p21) and Cyclin-Dependent Kinase Inhibitor 2A (CDKN2A, p16) (Supplementary Figure 2B) [30, 31]. These results suggest that the foot and trunk fibroblasts in WS have distinct gene expressions that regulate mesenchymal-lineage differentiation, but these differences are independent of cellular senescence.

### Foot fibroblasts in WS were less capable of adipogenesis compared with the trunk

**Figure 3:**
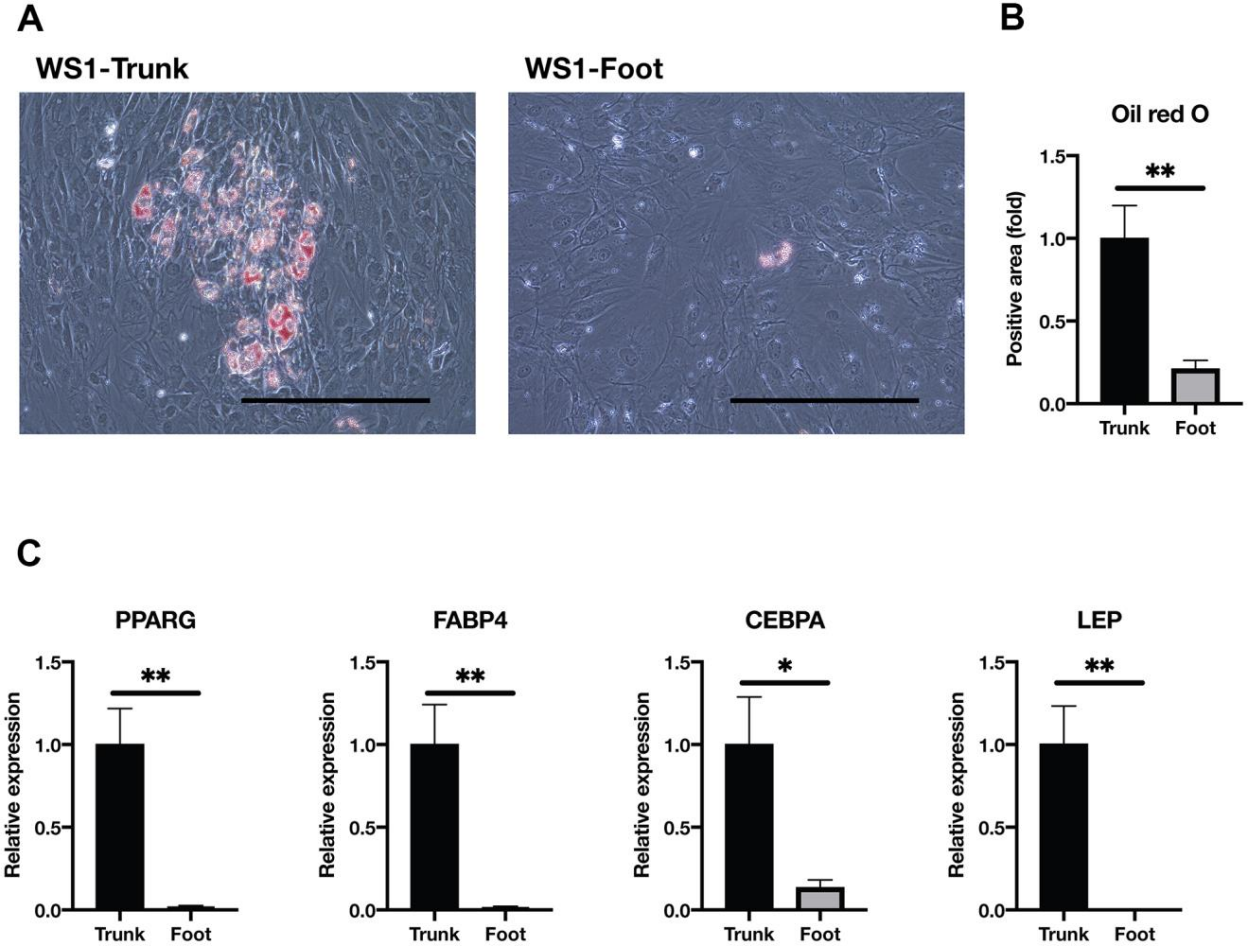
**Adipogenesis was impaired in the foot fibroblasts.** (**A**) Representative images of Oil red O staining after two weeks induction of adipogenesis in the trunk and foot fibroblasts of WS1. Bar = 300 μm. (**B**) Quantification of relative Oil red O staining area. Data are means ± SEM of two patients from four microscopic views. For statistical analysis, student t-test was performed (**p<0.01). (**C**) Relative gene expression analyzed by qRT-PCR. Data are means ± SEM of two patients with three technical replicates. For statistical analysis, student t-test was performed (*p<0.05; **p<0.01).

WS patients present with subcutaneous lipoatrophy in the extremities, while subcutaneous fat tends to accumulate in the trunk. Several reports have previously shown that human dermal fibroblasts can differentiate into mesodermal lineages *in vitro*, including adipocytes [32–34]. Taken together with the above results, we hypothesized that the adipogenesis potential is lower in foot fibroblasts than in the trunk. Thus, we investigated the adipogenic capacity of WS fibroblasts by culturing them in the adipogenic differentiation medium. After induction of adipogenesis, Oil red O staining results showed foot fibroblasts with significantly fewer oil droplets than the trunk (Figure 3A, 3B and Supplementary Figure 3). In gene expression analysis by qRT-PCR, adipocyte marker genes, PPARG, Fatty Acid Binding Protein 4 (FABP4), CCAAT Enhancer Binding Protein Alpha (CEBPA), and Leptin (LEP) were significantly decreased in the foot group compared with the trunk (Figure 3C) [35]. These results indicate that the trunk fibroblasts of WS maintain adipogenic capacity but the foot fibroblasts do not.

### Foot fibroblasts in WS exhibited an attenuated capacity for chondrogenesis

**Figure 4:**
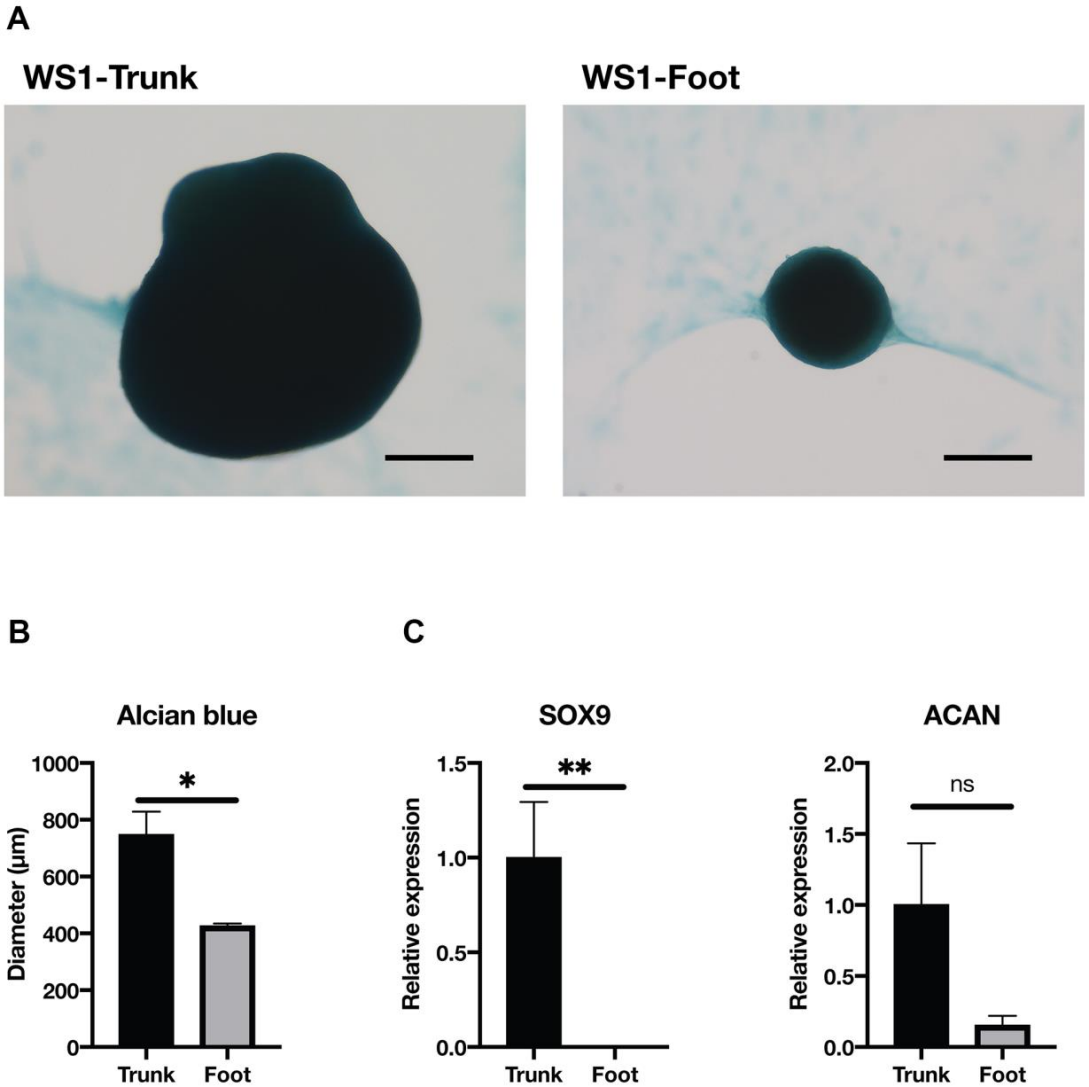
**Chondrogenesis tended to be reduced in the foot fibroblasts.** (**A**) Representative images of Alcian blue staining after two weeks induction of chondrogenesis in the trunk and foot fibroblasts of WS1. Bar = 300 μm. (**B**) Quantification of the diameter of chondrogenic spheroids. WS1-trunk, WS2-trunk, and WS1-foot were included. Data are means ± SEM. For statistical analysis, student t-test was performed (*p<0.05). (**C**) Relative gene expression analyzed by qRT-PCR. Data are means ± SEM of two patients with three technical replicates. For statistical analysis, student t-test was performed (ns, not significant; *p<0.05; **p<0.01).

Next, we performed chondrogenic differentiation to confirm a disparity in chondrogenesis between the trunk and foot fibroblasts. After the induction of chondrogenesis using the pellet method, the spheroid diameter was significantly smaller in the foot fibroblasts from WS1 than in the trunk group (Figure 4A, 4B). On the other hand, WS2 foot fibroblasts failed to maintain spheroid morphology (Supplementary Figure 4). The chondrogenesis differentiation marker, SRY-Box Transcription Factor 9 (SOX9), was significantly decreased in the foot group, and this tendency was also observed for Aggrecan (ACAN) (Figure 4C) [36]. These results suggest that WS foot fibroblasts tend to have a reduced capacity for chondrogenic differentiation compared with the trunk.

### Foot and trunk fibroblasts in WS were equally capable of osteogenesis

**Figure 5:**
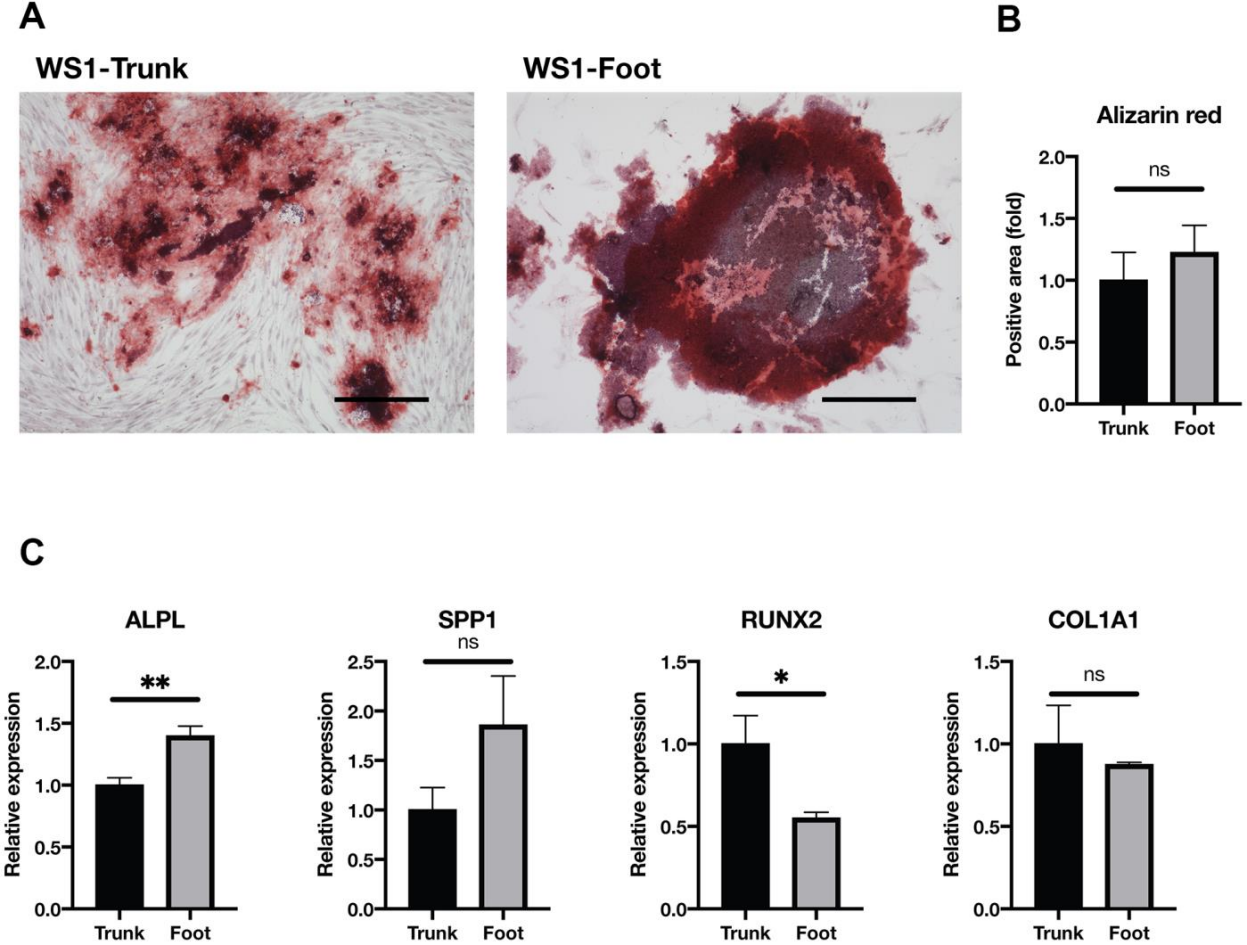
**Osteogenesis was maintained in both groups.** (**A**) Representative images of Alizarin red staining two weeks after induction of osteogenesis in the trunk and foot fibroblasts of WS1. Bar = 300 μm. (**B**) Quantification of relative stained area with Alizarin red. Data are means ± SEM of two patients with four microscopic views. For statistical analysis, student t-test was performed (ns, not significant). (**C**) Relative gene expression analyzed by qRT-PCR. Data are means ± SEM of two patients with three technical replicates. For statistical analysis, student t-test was performed (ns, not significant; *p<0.05; **p<0.01).

Next, the osteogenic differentiation ability was compared. After culturing in the osteogenesis medium, no clear difference was observed in the Alizarin red-stained area between the trunk and foot groups (Figure 5A, 5B and Supplementary Figure 5). The expression of Alkaline Phosphatase, Biomineralization Associated (ALPL), a marker of osteogenesis, was significantly elevated in the foot group (Figure 5C). On the other hand, RUNX Family Transcription Factor 2 (RUNX2) expression was significantly elevated in the trunk group, and there were no significant differences in other differentiation markers (Secreted Phosphoprotein 1, SPP1; Collagen Type I Alpha 1 Chain, COL1A1) (Figure 5C) [37]. These results suggest that foot fibroblasts in WS maintain the equivalent level of osteogenic differentiation capacity to the trunk.

## DISCUSSION

This is the first report comparing the phenotypes of dermal fibroblasts taken from different parts of the body of the same WS patient. The WS foot fibroblasts showed a reduced proliferative capacity with shorter telomeres, in comparison to the trunk fibroblasts. Transcriptome analysis showed increased gene expression related to osteogenic differentiation in the foot group and that of adipogenic and chondrogenic differentiation in the trunk group. Indeed, *in vitro* induction of adipogenesis and chondrogenesis of the foot fibroblasts showed significantly reduced differentiation capacity compared with the trunk. However, there was no difference in osteogenic capacity between the trunk and foot fibroblasts.

Previously, Rinn et al. conducted transcriptome analyses among normal human fibroblasts taken from different sites in the body [17, 18]. Among the DEGs between the extremities and trunk, the expression distribution of HOX genes is consistent with cell migration during human development [38]. Rinn et al. identified that HOXA13 was explicitly up-regulated in foot-derived fibroblasts, while the HOXB gene cluster was trunk-specific [17, 18]; these data are consistent with our results.

However, most of the genes we extracted through the DEG analysis in this study did not show site-specific changes in the previous reports [17, 18]. PPARG, which we found to be up-regulated in the trunk fibroblasts of the WS patients, is a master regulator of adipogenesis, and its overexpression promotes adipose differentiation [39]. In this study, the WS trunk-derived fibroblasts showed higher PPARG expression than the foot-derived ones, and there was a clear difference in the *in vitro* adipose differentiation ability. These apparent discrepancies are reminiscent of WS phenotypes, namely the trunk with relatively abundant subcutaneous fat and the extremities with lipoatrophy [15, 16]. In addition, although the anatomic origin of the fibroblast is unclear, WRN-depleted fibroblasts exhibit upregulation of PPARG [40]. Taken together with our findings, these results suggest that the regulation of PPARG gene expression in WRN-depleted cells might be context-dependent and that they can be down-regulated in the fibroblasts in the disease site. Further research is needed to understand the mechanism of downregulation of PPARG in WS foot fibroblasts compared to the WS trunk-derived fibroblasts. In addition, STMN2, a marker of osteogenesis, which is up-regulated during the osteogenic induction of mesenchymal stem cells [19], is the gene with the most distinct regulation in this study: the expression was overwhelmingly increased in the foot fibroblasts compared with the trunk.

Honjo et al. reported that painful subcutaneous calcification precedes skin ulcers in WS patients [13]. Patients with WS frequently suffer painful clavus and callus on the feet, which leads to the development of intractable skin ulcers [1, 12]. Ectopic soft tissue calcification also occurs in the limbs of WS patients [41]. Considering our findings that WS foot fibroblasts have a diminished ability to differentiate into adipocytes and chondrocytes while their osteogenic differentiation capacity remains fully preserved, the ossification of fibroblasts in the dermal and subcutaneous layers of the skin may result in these symptoms. In addition, reversible direct conversion of subcutaneous adipocytes into fibroblasts, *in vivo*, has been reported [42–44]. Therefore, our results suggest the possibility that subcutaneous lipoatrophy in WS extremities might attribute to the inability of adipogenic differentiation in fibroblasts in the disease site.

In this study, we revealed the distinct gene expression profiles and phenotypes in WS dermal fibroblasts derived from the foot and trunk. This study highlights the relationship between fibroblast phenotypes and WS-specific symptoms, refractory skin ulcers and subcutaneous lipoatrophy in extremities. These results could lead to a further understanding of the disease’s mechanism and development of a new therapeutic strategy in the future.

## MATERIALS AND METHODS

### Establishment of fibroblasts and cell culture

WS dermal fibroblasts were established from two WS patients (WS1 and WS2, Supplementary Table 4). Both were hospitalized for treatment of their foot skin ulcers, and the skin graft was taken from the trunk (groin). The healthy skin neighboring the ulcer and the skin partly taken from the graft were explanted into a dish as previously described [45]. Cell culture was performed with DMEM (043-30085, Wako, Osaka, Japan), supplemented with 10% FBS (10270106, Gibco, Waltham, MA, U.S.A), and antibiotic-antimycotic (15240062, Gibco) in humidified 5% CO_2_ air. The medium was changed every two days. When reaching sub-confluency, cells were passaged at a 1:4 split ratio until growth arrest and population doublings were calculated.

### Telomere quantitative fluorescence *in situ* hybridization (Q-FISH)

Fibroblasts at PD10 were treated with the colcemid kit (Chromocenter, Tottori, Japan) and fixed in Carnoy’s solution following the manufacturer’s protocol. The fixed cells on coverslips were treated with ribonuclease (312-01931, Nippon Gene, Tokyo, Japan) and 0.005 % pepsin (V1959, Promega, WI, U.S.), hybridized with peptide nucleic acid oligonucleotide probes (F1002, Panagene, Daejeon, South Korea), and immuno-stained with Hoechst 33342 (H342, Dojindo, Kumamoto, Japan), according to the manufacturer’s protocols. Images were recorded using a BZ-X700 microscope (Keyence, Osaka, Japan). Quantification was performed using the Telometer (https://demarzolab.pathology.jhmi.edu/telometer/), as previously described [46, 47].

### Quantitative polymerase chain reaction (qPCR)

RNA was extracted and reverse-transcribed, as previously described [48]. TaqMan Gene Expression Assays for PPARG (Hs01115513_m1), FABP4 (Hs01086177_m1), CEBPA (Hs00269972_s1), LEP (Hs00174877_m1), SOX9 (Hs00165814_m1), ACAN (Hs00153936_m1), ALPL (Hs01029144_m1), SPP1 (Hs00959010_m1), RUNX2 (Hs01047973_m1), COL1A1 (Hs00164004_m1), and B2M (Hs00187842_m1) were purchased from Applied Biosystems (Waltham, MA, U.S.). Quantification was performed with the ⊿⊿Ct method using B2M as an internal control.

### Tri-lineage differentiation

*In vitro* differentiation potentials of the fibroblasts into three mesenchymal lineages were evaluated by using adipogenesis, chondrogenesis, and osteogenesis differentiation kits (A1007001, A1007101, and A1007201, respectively; Gibco) according to manufacturer's protocols. Cells at PD 9 to 10 were used. After two weeks of differentiation, cells were sampled and stained. For each staining assay, Oil red O, Alcian blue, and Alizarin red staining (Sigma-Aldrich, St. Louis, MO, U.S.A) were used, respectively. Quantification of the stains was performed using a BZ-X700 microscope (Keyence, Osaka, Japan).

### Transcriptome analysis

mRNA was extracted from fibroblasts at PD 10 and the cDNA library was synthesized using the NEBNext Ultra RNA Library Prep Kit (E7370S, New England BioLabs, Beverly, MA, U.S.A). Sequencing was carried out (technically n=2 in each sample) by HiSeq1500 (Illumina, San Diego, CA, U.S.A) with 60bp single-reads. The reference genome mapping (UCSC/hg19) was performed using TopHat (version 2.0.13; with default parameters) with annotation data from iGenomes (Illumina). Cuffdiff (Cufflinks version 2.2.1; with default parameters) was used to quantify the gene expression levels. FPKM data were analyzed by iDEP (http://bioinformatics.sdstate.edu/idep/) as described by the authors [49].

### Study approval

All experiments were approved by the institutional review boards at the Chiba University Graduate School of Medicine (Chiba, Japan). Written informed consent was obtained from study participants before the commencement of this research.

## Supplementary Material

Supplementary Figures

Supplementary Table 1

Supplementary Table 2

Supplementary Table 3

Supplementary Table 4
