# Supplementary material for: Fibroblasts from different body parts exhibit distinct phenotypes in adult progeria Werner syndrome
Source: Aging (Albany NY). 2021 Feb 24;13(4):4946–61. doi: 10.18632/aging.202696 (PMC7950285; doi:10.18632/aging.202696)
Supplement: Supplementary Figures [file aging-13-202696-s001.pdf]

SUPPLEMENTARY FIGURES

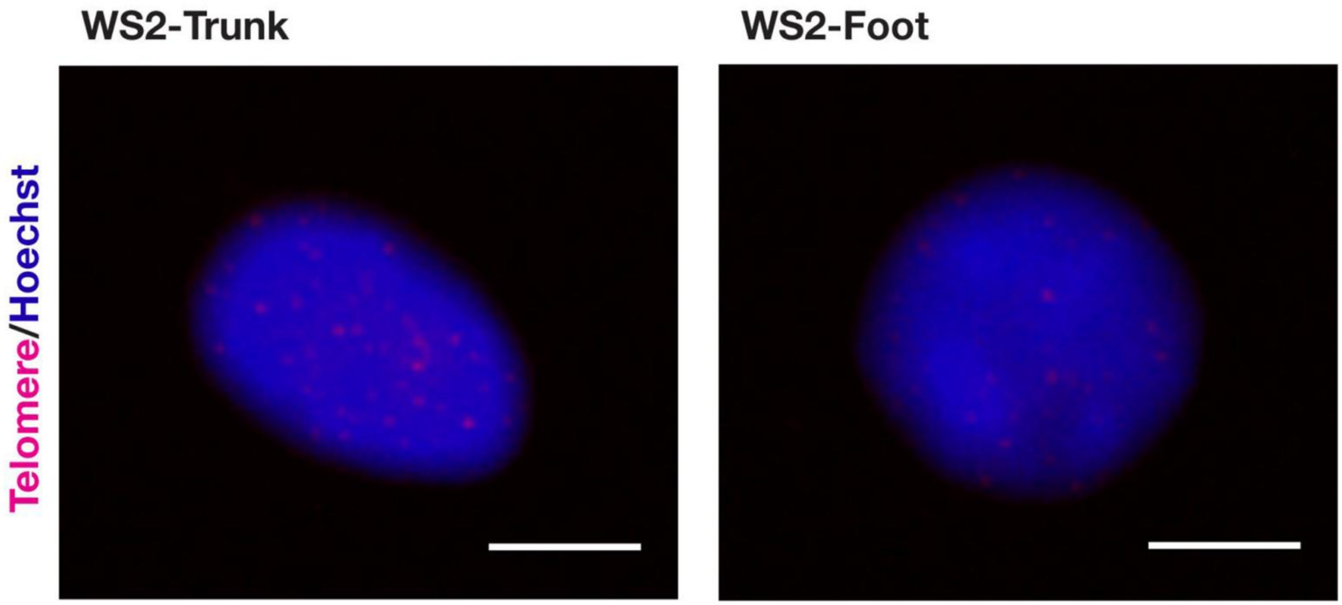

Supplementary Figure 1. Representative image of telomere Q-FISH of WS2. Bar = 10 µm.

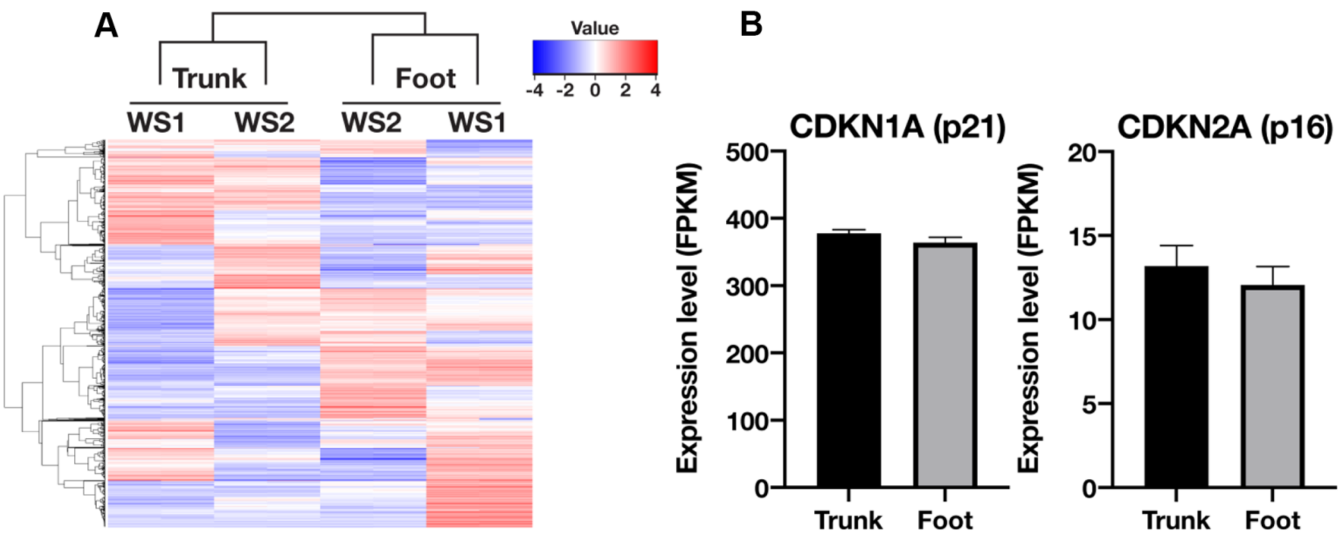

Supplementary Figure 2. Results of transcriptome analysis of the trunk and foot fibroblasts. (**A**) Heatmap of the hierarchical clustering analysis. (**B**) FPKM results of CDKN1A (p21) and CDKN2A (p16). Data are means ± SEM of two patients (technically n=2 in each sample).

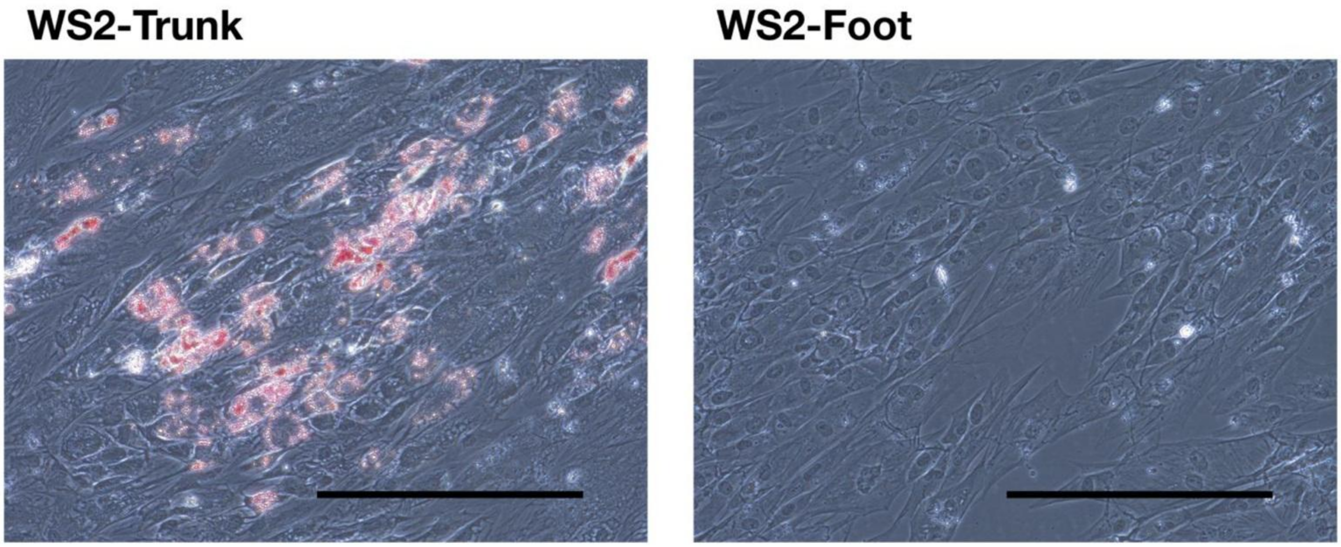

Supplementary Figure 3. Representative images of Oil red O staining two weeks after induction of adipogenesis in the trunk and foot fibroblasts of WS2. Bar = 300 μm.

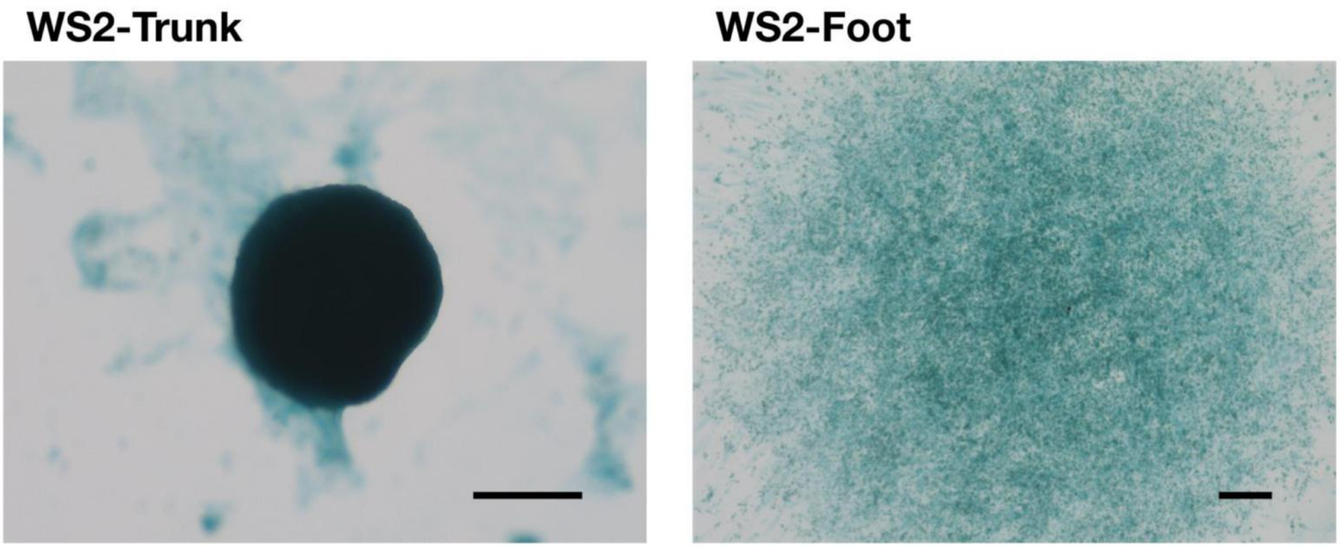

Supplementary Figure 4. Representative images of Alcian blue staining two weeks after induction of chondrogenesis in the trunk and foot fibroblasts of WS2. Bar = 300 μm.

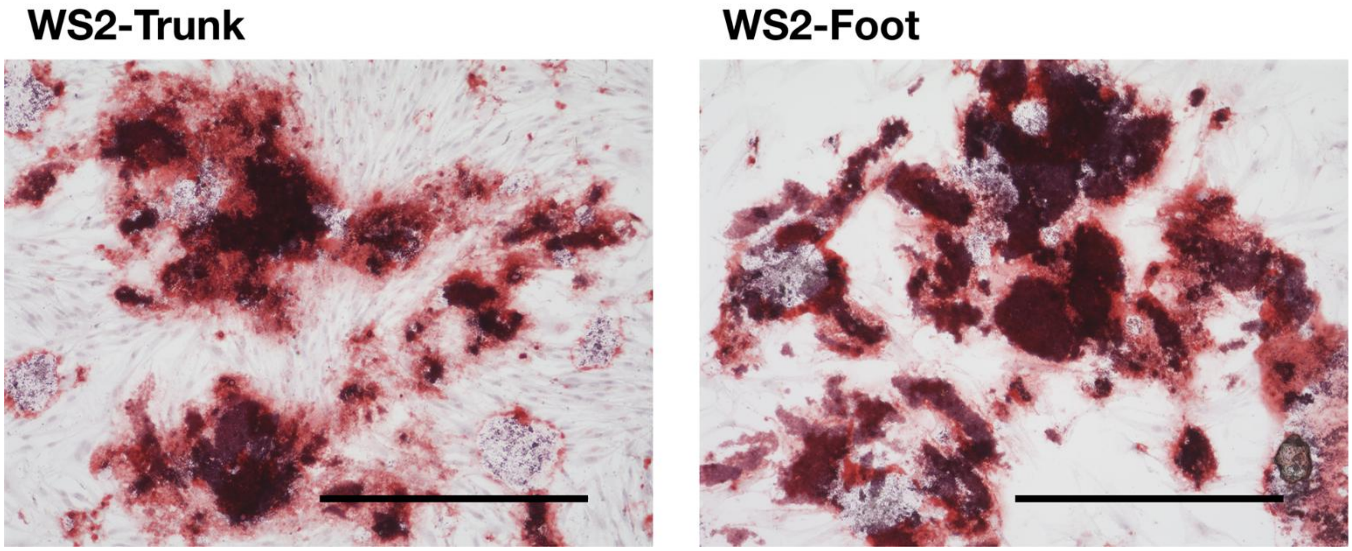

Supplementary Figure 5. Representative images of Alizarin red staining two weeks after induction of osteogenesis in the trunk and foot fibroblasts of WS2. Bar = 300 µm.
